# A multiphase, multimethod protocol for evaluating air-in-line alarms and infusion-technology usability during antineoplastic drug administration

**DOI:** 10.1016/j.mex.2026.104180

**Published:** 2026-09-26

**Authors:** Rosario Caruso, Greta Ghizzardi, Tatiana Bolgeo, Monica Liberatori, Daniele Napolitano, Anna Carmagnola, Andrea Moro, Ilaria Milani, Stefano Maiandi, Gianluca Conte, Marco Alfredo Arcidiacono, Serena Caponetti, Gabriele Cremona, Marcella Dabbene, Viviana Riccobene, Monica Guberti, Alessio Piredda, Arianna Magon

**Affiliations:** a Department of Biomedical Sciences for Health, University of Milan, Milan, Italy; b Health Professions Research and Evidence Transfer Unit, IRCCS MultiMedica, Sesto San Giovanni, Italy; c School of Nursing, Directorate of Nursing and Allied Health Professions, Azienda Socio-Sanitaria Territoriale di Lodi, Lodi, Italy; d Research Training Innovation Infrastructure, Department of Research and Innovation, AOU SS Antonio e Biagio e Cesare Arrigo, Alessandria, Italy; e Department of Medical Oncology, Fondazione Policlinico Gemelli IRCCS, Rome, Italy; f SITRA - Scientific Direction, Fondazione Policlinico Gemelli IRCCS, Rome, Italy; g Hematology and Bone Marrow Transplantation Unit, IRCCS San Raffaele Scientific Institute, Milan, Italy; h Directorate of Nursing and Allied Health Professions, European Institute of Oncology IRCCS, Milan, Italy; i Department of Medicine and Surgery, Kore University of Enna, Enna, Italy; j Medical Department, University Hospital of Parma, Parma, Italy; k Oncology Department, Ospedale “A. Murri”, AST, Fermo, Italy; l Department of Hematology-Oncology, Piacenza Hospital, Piacenza, Italy; m Nursing Management, La Maddalena S.p.A., Palermo, Italy; n Oncology Ward, Presidio Ospedaliero "Gravina e San Pietro", ASP di Catania, Caltagirone, Italy; o Nursing and Allied Health Professions, IRCCS Istituto Ortopedico Rizzoli, Bologna, Italy; p Outpatient Service, IEO, European Institute of Oncology IRCCS, Milan, Italy; q Department of Public Health, Experimental and Forensic Medicine, Section of Hygiene, University of Pavia, Pavia, Italy

**Keywords:** Infusion pumps, Antineoplastic agents, Patient safety, Alarm management, Simulation training, Multiphase study design, Multimethod research

## Abstract

Air-in-line alarms during intravenous administration of antineoplastic drugs can interrupt therapy, increase nursing workload, and affect the continuity and safety of treatment. Although advanced infusion technologies have been introduced to reduce these events, evidence comparing their performance in clinical practice remains limited. This protocol describes a multicenter, prospective, multiphase study, combining a controlled simulation phase with two observational phases, designed to investigate the occurrence and management of air-in-line alarms during antineoplastic drug administration. The study aims to identify technological, clinical, and organizational factors associated with alarm occurrence and management while evaluating healthcare professionals’ user experience and observed professional practices related to infusion therapy. Findings will support the development of evidence-based recommendations to improve infusion safety, optimize therapeutic continuity, and guide the selection and implementation of infusion technologies in oncology settings.

## Graphical abstract

**Figure:**
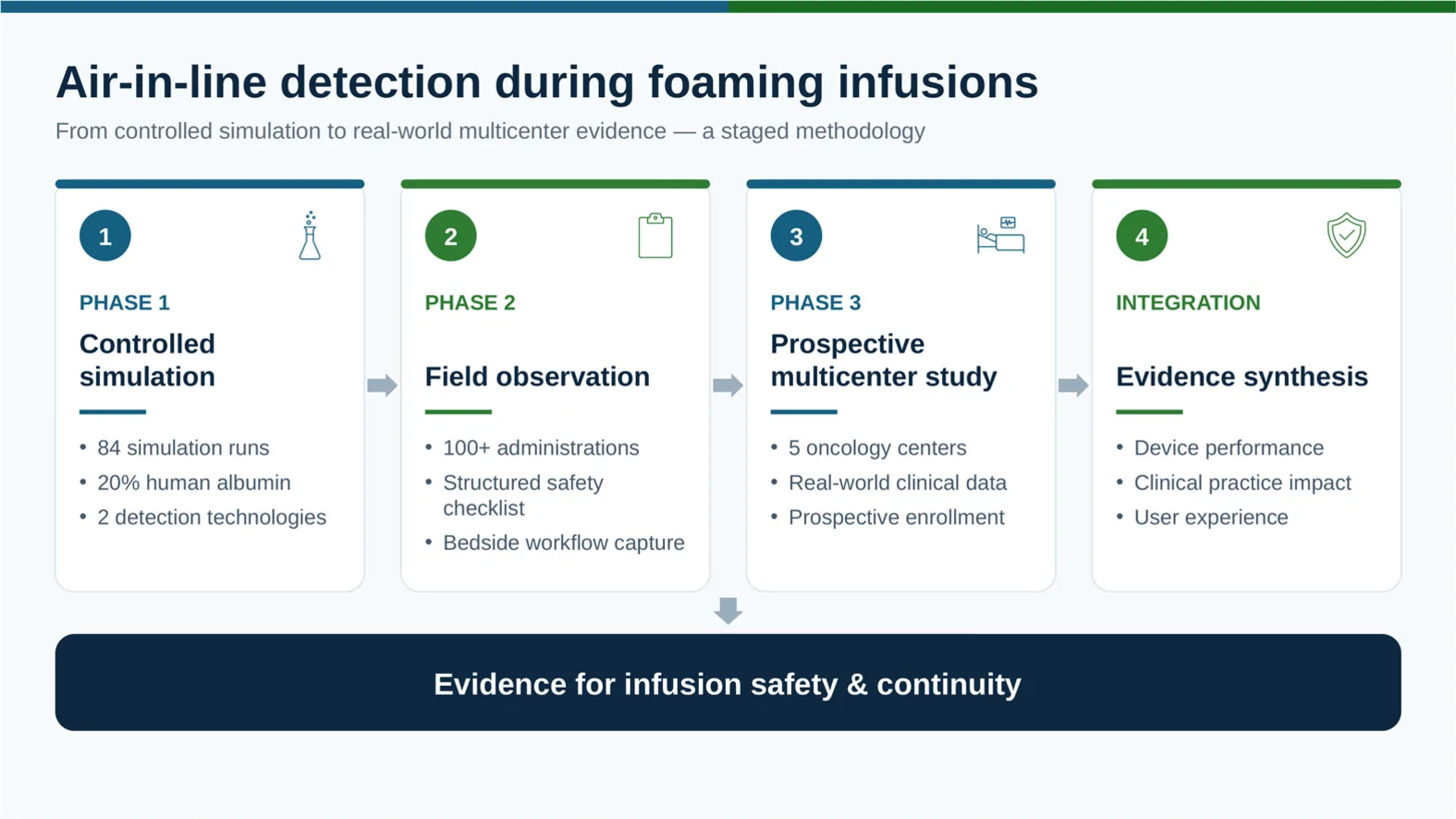

## Specifications table

**Table:** 

**Subject area**	Medicine and Dentistry
**More specific subject area**	Oncology Nursing
**Name of your protocol**	A multiphase, multimethod protocol for evaluating air-in-line alarms and infusion-technology usability during antineoplastic drug administration
**Reagents/tools**	Controlled bench-simulation infusion setup; standardized foaming preparation (20% human albumin, 200 g/L); cassette-based, piston-actuated positive-displacement volumetric infusion technology; and tubing-based linear-peristaltic volumetric infusion technology.
Experimental design	Multicenter, prospective, multiphase, multimethod study comprising three sequential and partially overlapping phases: a controlled bench-simulation study; a field observational study applying an Entrustable Professional Activities (EPA)-informed framework; a prospective multicenter observational cohort study comparing infusion technologies in real-world clinical practice
**Trial registration**	NCT07718711
**Ethics**	Ethical approval was obtained from the Italian National Ethics Committee (Comitato Etico Nazionale, CEN: AOO-ISS - 04/03/2026 – 0009,745 on July 20, 2026). Phase 1 will not involve personal data. In Phases 2 and 3, personal data of participating healthcare professionals will be processed in minimized, coded form, after written informed consent, and reported only in aggregate, in compliance with the GDPR and Italian legislation. No clinical data referable to patients will be collected at any phase.
**Value of the Protocol**	• This protocol describes a multiphase, multimethod design integrating simulation, field observation, and real-world clinical data to study air-in-line alarms during antineoplastic drug administration, an area with limited prospective frequency estimates and independent comparative evidence.
	• The protocol applies an Entrustable Professional Activities (EPA)-informed observation framework to describe professional practices relevant to safe infusion and alarm management, building on recent consensus-based development of EPAs for anticancer therapy nursing.
	• Findings will support clinical practice and the development of evidence-based recommendations for infusion technology selection, alarm management strategies, and training pathways for healthcare professionals.

## Background

Antineoplastic drugs are high-alert medications: their toxicity and narrow therapeutic index mean that administration errors can cause serious patient harm, and errors occur at every stage of the medication-use process [[1], [2], [3]]. Their handling also entails occupational risks for healthcare professionals, requiring appropriate safeguards throughout preparation and administration [4,5].

Air-in-line alarms are among the most frequent infusion-pump alarms; they interrupt drug delivery, delay treatment, and increase nursing workload [[6], [7], [8], [9]]. In large multicenter datasets, antineoplastic agents and monoclonal antibodies generate a disproportionate share of air-in-line alarms, and oncology units show the highest air-in-line alarm rates [7,8,10]. Alarm occurrence depends on solution characteristics, circuit preparation, administration sets, sensor settings, and the core pumping technology [9,[11], [12], [13]]. Solutions prone to foaming or outgassing, including albumin-containing formulations, can generate microbubbles that trigger repeated air-in-line alarms [12,14], and manual air removal frequently requires disconnection or aspiration of hazardous-drug circuits [12]. Nevertheless, prospective estimates of air-in-line alarm incidence during antineoplastic infusions remain scarce, and independent comparisons of infusion technologies under standardized conditions are lacking [9].

Large-volume infusion systems differ in their pumping and air-management architecture. A cassette-based, piston-actuated positive-displacement volumetric system delivers a defined volume per cycle by deflecting a diaphragm within a cassette pumping chamber and may integrate actively controlled valves, proximal and distal air detection, an air trap, and backpriming; a tubing-based linear-peristaltic volumetric system advances fluid through sequential compression of the administration tubing [12,13]. These differences involve the complete infusion system, including the pump mechanism, administration set, and sensing functions, rather than the disposable set alone. Whether they reduce clinically disruptive alarms during infusions with foaming solutions has not been independently established [9,12].

Technology performance alone does not determine safe implementation: usability, alarm design, workload, and user experience influence workarounds and adherence to safe practice [[15], [16], [17]]. Professional competencies are equally central to alarm recognition, circuit management, and restoration of infusion continuity. Entrustable Professional Activities (EPAs) translate competencies into observable units of professional work [18,19]; they are increasingly used in nursing [20], and EPAs for systemic anticancer therapy nursing have recently been developed through expert consensus [21].

Evidence on these points therefore remains fragmented. Head-to-head comparisons of pumping architectures under standardized foaming conditions are lacking; the frequency and time cost of air-in-line alarms in routine oncology care have rarely been quantified prospectively [9]; recently developed EPAs for anticancer therapy nursing require evaluation in workplace practice [21]; and usability is seldom measured alongside observed practice [15]. Consequently, the relative weight of technological, clinical, and organizational determinants of effective alarm management remains unknown, and practical recommendations rest on limited evidence [9,12]. This protocol addresses these gaps through a multiphase, quantitative multimethod design integrating controlled simulation, structured field observation, and prospective real-world clinical observation.

### Description of protocol

#### Overall objective

To evaluate the technological, clinical, and organizational factors associated with the occurrence and management of air-in-line alarms during intravenous antineoplastic drug administration, thereby informing evidence-based strategies to improve infusion safety and therapeutic continuity.

#### Specific objectives

• To compare, in a controlled simulation environment, the occurrence of air-in-line alarms between a cassette-based, piston-actuated, positive-displacement volumetric system and tubing-based linear-peristaltic volumetric systems using a standardized foaming preparation.
• To describe, through structured field observation using an EPA-informed framework, professional practices relevant to safe management of infusion therapy with antineoplastic drugs.
• To analyze the frequency, type, and management times of infusion alarms in real-world clinical settings.
• To evaluate healthcare professionals’ user experience with different infusion technologies.
• To identify clinical, technological, and organizational factors associated with effective management of air-in-line alarms.
• To support the development of operational recommendations for optimizing clinical practice and training pathways for healthcare professionals.

#### Primary outcomes

The primary outcomes are phase-specific: at least one air-in-line alarm per simulation run in Phase 1; at least one air-in-line alarm or qualifying unplanned infusion interruption per observed administration in Phase 2 (a composite outcome, with components reported separately); and at least one air-in-line alarm per eligible administration in Phase 3. Operational definitions are provided in the corresponding Methods sections.

#### Secondary outcomes

Secondary outcomes, as specified for each phase, include alarm counts and types, alarm-management time, operational strategies, adherence to evidence-based infusion practices, healthcare professionals’ user experience, variability across centers and clinical settings, and factors associated with alarm occurrence and management.

The phase-specific primary outcomes follow the approved protocol. The public registration (NCT07718711) describes alarm frequency and type as a broad primary outcome; here, the binary primary endpoints are distinguished from secondary alarm counts and types. Alarm-management time and operational strategies remain secondary. The registered user-experience description is broader than the UEQ: only its six defined scales will be interpreted as UEQ outcomes, with any supplementary items reported separately. The registered enrollment of 40 professionals and the Phase 3 target of 200 administrations refer to different units of observation.

### Importance of the protocol

Air-in-line alarms are a frequent but under-measured source of disruption in antineoplastic infusion, with consequences for treatment continuity, nursing workload, and occupational safety. The importance of this protocol lies in examining the problem at three complementary levels within a single prespecified framework: device behavior under standardized foaming conditions, observable nursing practice during routine care, and real-world alarm frequency, management time, and user experience across multiple centers. This integration allows technological, professional, and organizational determinants to be interpreted together rather than in isolation, as has mostly been the case so far. The protocol also makes its own boundaries explicit: comparisons concern technology–device configurations as approved for clinical use, technology–center alignment is declared a priori, and planning proportions are treated as scenario-based assumptions. The resulting estimates will inform infusion-technology selection, alarm-management procedures, and competency-based training in oncology nursing, and the prespecified instruments and analysis plan can be adapted to other infusion technologies, drug classes, and healthcare systems.

### Study design

This protocol describes a multicenter, prospective, multiphase, quantitative multimethod study comprising a controlled simulation phase and two prospective observational phases. The three phases use distinct units of observation and complementary data sources to examine the technological, professional, and organizational dimensions of air-in-line alarm occurrence and management during antineoplastic drug administration.

Partial disclosure. To limit observation reactivity and the Hawthorne effect, a partial-disclosure procedure approved by the CEN will be used in Phases 2 and 3. Participants will receive all information required for valid consent; only the specific analytical hypotheses and the professional behaviors under assessment will be temporarily withheld. Participants will be debriefed after the observation period and may request withdrawal of their data until these have been irreversibly anonymized or incorporated into aggregate analyses.

**Phase 1:** controlled simulation study. The first phase will compare the occurrence, number, and time to onset of air-in-line alarms between a cassette-based, piston-actuated positive-displacement volumetric system and tubing-based linear-peristaltic volumetric systems under standardized conditions, using a standardized foaming preparation. It will involve neither patients nor healthcare-professional participants.

**Phase 2:** prospective field-observation study. The second phase will use structured, EPA-informed observation of routine antineoplastic infusions to characterize professional practices related to infusion preparation, device management, alarm recognition and resolution, and restoration of infusion continuity.

**Phase 3:** prospective multicenter observational cohort study. The third phase will observe antineoplastic administrations performed in routine practice with the infusion technology already in use at each center, recording alarm occurrence, type, and management time, management strategies, and nursing professionals’ user experience. No technology will be assigned and no modification of standard care will be introduced.

**Integration across phases.** The three phases have different units of observation and will be analyzed separately, without pooling or participant-level linkage between Phases 2 and 3. Findings will be integrated at the interpretation stage through a prespecified cross-phase framework linking technology performance, observed professional practice, real-world alarm outcomes, and user experience; convergence and discrepancies will be examined in relation to device configuration, center, clinical context, and workflow.

**Local study coordination.** Each center will appoint a local study coordinator, preferably a nursing coordinator, to support protocol implementation, observer training, data-collection logistics, and completeness checks. Where feasible, coordinator and observer will be different individuals. Coordinators will not assign technology, alter routine practice, modify recorded data, adjudicate outcomes, or perform the primary analysis.

## Methods

For the controlled bench-simulation phase, applicable reporting items from the simulation-research guidance [22] will be adapted to describe the equipment, procedures, outcomes, replication, and limitations. The prospective observational phases will be reported according to the STROBE Statement [23]; full adherence to an observational-study checklist is not claimed for the device-only experiment.

### Phase 1 – Controlled simulation study

#### Study design and setting

Phase 1 will be a prospective, multicenter, controlled bench-simulation study conducted in three simulation laboratories; no device will be connected to a patient. Should any site prove unable to provide analyzable data, this will be reported, and all Phase 1 findings will be explicitly referred to the contributing sites.

The study will compare the cassette-based, piston-actuated positive-displacement volumetric system (Device A) with the tubing-based linear-peristaltic volumetric systems (Devices B and C) described in the Background and in Table 1. The pump mechanism, administration set, air-detection system, and air-management features will be treated as integrated components of the tested technology rather than as independent experimental factors, and only administration sets approved for the corresponding infusion system will be used.

**Table 1:** Infusion devices.

Device code	Technological classification	Role in the study	Study phases
Device A	Cassette-based, piston-actuated positive-displacement volumetric infusion system	Technology under comparative evaluation	Phases 1 and 3
Device B	Tubing-based linear-peristaltic volumetric infusion system	Comparator technology	Phases 1 and 3
Device C	Tubing-based linear-peristaltic volumetric infusion system	Comparator technology	Phases 1 and 3

Each technology will be evaluated at every site where available, with run order randomized within site so that technology, device model, site, and operator effects can be distinguished as far as the design allows; each physical pump unit will be identified. The numbers of independent pump units and runs per unit, model, operator, and site will be specified before formal simulations and reported separately. Configurations available at only one site will be documented and their comparisons considered exploratory. Phase 1 is not designed to establish the general superiority of a technology class.

#### Standardized simulation procedure

Each analyzable simulation will consist of the infusion of 50 mL of 20% human albumin solution at a programmed flow rate of 100 mL/h. Human albumin was selected as a protein solution with recognized foaming propensity, a property also described for albumin-containing antineoplastic formulations [14]. Before formal simulations, a versioned standard operating procedure will specify solution handling and foam generation, agitation and settling times, priming, ambient-temperature range, and set replacement or reuse between runs. Circuit preparation procedure, room temperature, presence of visible foam or bubbles before infusion, activation of the air-in-line detection system, and completion of the programmed volume will be recorded for each run. Training and calibration runs will be identified prospectively in the electronic case-report form and excluded from the primary analysis. Runs will be considered analyzable when classified as data-collection sessions containing sufficient information to determine the primary outcome.

#### Pressure and air-detection settings

The downstream occlusion-alarm limit will be harmonized at approximately 200 mmHg: 4 psi (≈207 mmHg) for the cassette-based system and the closest manufacturer-available setting within 200 ± 25 mmHg for each tubing-based system. The exact numeric setting used in each run will be recorded.

Occlusion-pressure and air-in-line detection thresholds will be treated as separate parameters; harmonized occlusion pressure does not imply equivalent air-detection sensitivity. Air-in-line detection will remain enabled according to the manufacturer-approved setup, and the single-bubble and cumulative-air thresholds, detection window, default or customized sensitivity, software version, administration-set catalog number and lot, and pump-unit identifier will be recorded for every run. Where approved configurations do not permit numerically equivalent thresholds, this will be reported, and the comparison interpreted as one between technology–device configurations as approved for clinical use.

#### Primary and secondary outcomes

The primary outcome will be the occurrence of at least one air-in-line alarm during a simulation run, expressed as a binary variable.

Secondary outcomes will be the total number of air-in-line alarms per run, time from infusion initiation to the first air-in-line alarm, occurrence of other infusion alarms, need to interrupt the infusion, time required to manage an alarm, alarm recurrence before completion of the programmed volume, management strategies used, and successful completion of the infusion.

Alarm counts will not be treated as a continuous, normally distributed outcome. Alarm-management time will be evaluated only among runs in which a relevant alarm occurs and will be interpreted descriptively, because this subgroup is conditioned on the primary outcome.

#### Sample-size determination

No independent published study provides directly transferable estimates of the proportion of simulation runs with at least one air-in-line alarm during a standardized 20% albumin infusion. Proportions of 0.40 for tubing-based linear-peristaltic systems and 0.10 for the cassette-based, piston-actuated positive-displacement system have therefore been prespecified as scenario-based planning assumptions, informed by unpublished preliminary pilot data collected by the Italian Association of Oncology Nurses (AIIAO). Given the limited size and exploratory nature of the pilot dataset, these proportions inform sample-size planning rather than serving as estimates of alarm incidence [24].

Using the normal approximation for a two-sided comparison of two independent proportions, a significance level of 0.05, nominal 90% power, and equal allocation, the prespecified scenario of 0.40 versus 0.10 requires 42 analyzable simulations per group (84 overall). Under the same assumptions, more conservative effects require 57 simulations per group (0.35 vs 0.10), 65 (0.40 vs 0.15), 82 (0.30 vs 0.10), or 97 (0.35 vs 0.15). These are nominal planning values: exact-test discreteness and dependence among repeated runs can reduce power. Smaller differences may remain imprecisely estimated, and a nonsignificant result will not establish equivalence.

#### Statistical analysis

The primary analysis will compare the proportion of runs with at least one air-in-line alarm between the cassette-based system and the pooled tubing-based systems, using Pearson’s chi-square test, or Fisher’s exact test when expected counts are small. The risk difference, risk ratio, and odds ratio will be reported with 95% confidence intervals (two-sided α = 0.05). Outcomes will also be reported descriptively for each device-model configuration.

Alarm counts will be summarized using mean, standard deviation, median, interquartile range, range, and proportion of zero-alarm runs; any comparison will be exploratory, with a negative-binomial model only if events and overdispersion allow reliable estimation. Time to first alarm will be treated as right-censored at completion of the programmed infusion and summarized descriptively if events are few. Management time and strategies will be summarized only for alarm events and not formally compared when either group has fewer than five evaluable events.

Multilevel models or cluster-robust standard errors will be used only if the numbers of sites and pump units support reliable estimation; otherwise, results will be summarized by pump unit and configuration, and any run-level inferential analyses will be explicitly labeled exploratory with their independence assumption and will not support causal attribution to the core technology.

#### Data-quality procedures and sensitivity analyses

Device-model labels will be standardized while preserving original entries in the audit trail. Logical consistency checks will be applied to all outcome and process variables, and discordant records will undergo source-data verification before database lock. Unresolvable discrepancies will be classified as missing, and sensitivity analyses will test alternative classifications of the primary outcome; results will be considered robust only if their direction and interpretation are unchanged. No observation will be excluded because of its magnitude or influence, and all exclusions and corrections will be logged.

#### Interpretation and generalizability

Because repeated runs with the same site-specific technology–device configuration increase precision but do not replicate the core technology independently, results will be interpreted as evidence on the evaluated configurations under standardized conditions; generalization to technology classes would require multiple pump units per model, with both technologies tested at each site.

### Phase 2 – Prospective structured field-observation study

#### Study population and unit of observation

The study population will comprise nursing professionals involved in the preparation, administration, monitoring, or management of intravenous antineoplastic therapy in routine oncology settings. All eligible professionals working during the scheduled observation periods will be invited to participate and will provide written informed consent before being observed.

The primary unit of observation will be an eligible antineoplastic drug administration rather than the individual professional, who may therefore contribute more than one observed administration. Each consenting professional will be assigned a phase-specific pseudonymous code, so that repeated observations can be identified analytically without direct identifiers in the central research dataset.

An eligible administration will be defined as an intravenous antineoplastic infusion performed by a consenting nursing professional during a prespecified observation session and monitored from the initiation of the infusion procedure until completion, transfer of responsibility, or termination of the observation period. Actual observation duration, completion status, and the reason for ending observation will be recorded. An incompletely observed administration with no recorded event will not be classified as event-free for the entire infusion. Scheduled pauses, planned changes of infusion container, and routine end-of-infusion signals will not be classified as unplanned interruptions.

#### Sampling and sample-size rationale

Eligible administrations will be included consecutively during prespecified observation periods at the five participating centers, covering different days, time periods, clinical areas, and sessions where feasible, to reduce discretionary selection.

A minimum of 100 analyzable administrations is planned, based on precision rather than power: assuming that about 30% of administrations involve at least one qualifying event, under independence, 100 observations will estimate this proportion with a 95% confidence-interval half-width of approximately 9 percentage points. Clustering may widen this interval; the target supports descriptive estimation of event frequencies and practices, not prediction-model development.

The operational target will be approximately 20 analyzable administrations per center; deviations will be documented. With approximately eight eligible nursing professionals per center, up to about 40 professionals may contribute observations; this describes the available population and is not a participant-level sample-size calculation.

Administrations will be classified according to a prespecified list of solutions with or without recognized foaming or outgassing propensity, finalized before analysis by oncology-pharmacy and infusion-therapy experts and not modified after examination of outcomes.

#### Data sources and outcome definitions

Data will be collected through structured direct observation using a standardized electronic observation grid. The instrument will be developed from applicable evidence-based infusion practices and an Entrustable Professional Activities–informed framework adapted to antineoplastic infusion therapy.

The primary Phase 2 outcome will be the occurrence of at least one qualifying infusion-related event during an observed administration. A qualifying event will comprise an air-in-line alarm or an unplanned interruption attributable to the infusion system, administration set, solution characteristics, or alarm-management process. The individual components of this composite outcome will always be reported separately. If observation ends before infusion completion, an observed qualifying event establishes a positive outcome; otherwise, the full-administration binary outcome will be treated as unascertained. Time-to-first-event analyses will censor follow-up at the end of observation, and a sensitivity analysis will be restricted to fully observed administrations.

Secondary outcomes will include the number and type of alarms; time to first alarm; alarm-management time (from alarm activation or recognition to safe restoration of the prescribed infusion); alarm recurrence; manipulation or replacement of the administration set; maintenance of a closed circuit; operational strategies; and restoration of infusion continuity.

Infusion technology will be classified by its core pumping mechanism (Table 1) and treated as an observed exposure, not assigned by the study team. Device model, software version, administration-set characteristics, and air-detection and occlusion-pressure settings will be documented whenever available.

The observation grid will include predefined, directly observable behaviors mapped to EPA-informed professional-activity domains, each recorded as performed, not performed, not applicable, or not observable; the last two categories will be excluded from adherence denominators. The framework will describe observed performance and training needs and will not be used for entrustment decisions or to infer overall individual competence.

#### Observer training and bias control

Observers will complete standardized training on variable definitions, event timing, grid use, ambiguous situations, and non-interference with routine care, supported by a data dictionary and operational manual.

Where feasible, at least 10% of administrations will be assessed independently by two observers, with agreement quantified by percentage agreement and Cohen’s kappa for categorical variables and intraclass correlation coefficients for times. Because observers cannot be blinded to technology or to visible and audible alarms, classification bias will be limited through prospective definitions, structured fields, objective timestamps where available, and separation of observation from analysis.

#### Statistical analysis

Characteristics of participants, administrations, technologies, and centers will be summarized as counts and percentages with 95% confidence intervals, or means and standard deviations or medians and interquartile ranges, as appropriate. The proportion of administrations with at least one qualifying event will be reported overall, separately for air-in-line alarms and unplanned interruptions, and by center, foaming classification, clinical context, and technology. Right-skewed management times will be reported as medians, interquartile ranges, and ranges.

Comparative analyses will be exploratory. Binary outcomes will be examined using logistic regression with center as a fixed effect and a professional-level random intercept, where estimable. Center-adjusted technology associations will be estimated only if there is sufficient within-center technology variation; otherwise, technology comparisons will describe the observed technology–center configurations, and adjusted models will examine other covariates with sufficient within-center variation. If sparse events prevent reliable estimation, penalized logistic regression or stratified descriptive analyses will be used. Management time will be modeled only if the number of evaluable alarms is sufficient relative to model parameters.

Adherence will be calculated as the number of applicable behaviors performed divided by the number of applicable and observable behaviors, and its associations with prespecified variables (technology, foaming classification, clinical context, professional experience, center) will be assessed using models appropriate to its distribution and subject to the same identifiability restrictions as the binary-outcome analyses. The term “associated factors” rather than “predictive factors” will be used.

Effect estimates will be presented with 95% confidence intervals, and all tests will be two-sided (p < 0.05). Because subgroup and multivariable analyses are exploratory, interpretation will emphasize effect sizes, confidence intervals, consistency, and clinical relevance, and no causal effect of technology, professional experience, or organizational characteristics will be inferred.

Missingness will be reported for each principal variable; missing primary outcomes will not be imputed, and missing covariates will be handled by complete-case analysis when minimal or by an explicitly documented method otherwise.

### Phase 3 – Prospective multicenter observational cohort study

#### Study population and units of observation

Phase 3 will be conducted under routine clinical conditions at five oncology centers. No infusion technology, drug regimen, or clinical procedure will be assigned or modified by the study team. The cassette-based system (Device A) is in routine use at one participating center, whereas the tubing-based linear-peristaltic systems (Devices B and C) are in routine use at the remaining four; technology is therefore aligned with center by design (see Limitations).

Two units of observation will be used: the individual antineoplastic administration for alarm outcomes, and the consenting nursing professional evaluating a specific technology for user experience. The two levels will be analyzed separately and linked only through pseudonymous identifiers.

Eligible nursing professionals will be those directly involved in programming, initiating, monitoring, or managing antineoplastic infusions during the study period; each will provide written informed consent and receive a phase-specific pseudonymous code.

#### Sampling and sample size

Eligible administrations will be included consecutively within each center and technology stratum during prespecified data-collection periods covering different days, time periods, clinical areas, and sessions where feasible. An administration will be analyzable when both the infusion technology and the presence or absence of the primary outcome can be determined; non-analyzable administrations will be documented with reasons and not selectively replaced. Data-collection periods will accommodate an anticipated 10% of non-analyzable administrations, which will be verified prospectively.

The planning outcome will be the occurrence of at least one air-in-line alarm per administration. Assuming scenario-based planning proportions of 0.35 for tubing-based and 0.15 for cassette-based systems (informed by pilot data), a normal-approximation calculation with two-sided α = 0.05, nominal 90% power, independent observations, and equal allocation, approximately 97 administrations per group are required, rounded to a minimum of 100 analyzable administrations per group (200 overall).

Because these proportions are provisional, analysis will emphasize estimation: under independence, with 100 administrations per group, proportions of 0.15 and 0.35 would be estimated with 95% confidence-interval half-widths of about 7 and 9 percentage points, and a risk difference of 0.20 with a half-width of about 12 percentage points. These nominal intervals do not incorporate clustering. No operational recommendation will rest on a point estimate alone.

This calculation assumes independent observations, whereas the planned data will be clustered. Administrations will be clustered within professionals and centers, and because technology is aligned with center, the cassette-based group will form a single center cluster (about eight professionals) against four centers (about 32 professionals) in the tubing-based group. The groups will therefore carry different design effects, so the nominal 90% power is an optimistic planning benchmark rather than a guaranteed operating characteristic. Sensitivity analyses will use professional-level intracluster correlation coefficients of 0.01, 0.05, and 0.10 as illustrative planning scenarios. At 100 administrations per group and approximately 8 versus 32 professionals, the equal-cluster-size approximation DE = 1 + (m − 1)ρ gives design effects of 1.12, 1.58, and 2.15 for the cassette-based group and 1.02, 1.11, and 1.21 for the tubing-based group. These scenarios quantify professional-level dependence only; they do not estimate the center-level component for the single cassette-based center or remove technology–center confounding.

The target of 100 analyzable administrations per group will therefore be treated as a minimum rather than a stopping rule, and recruitment will continue throughout the prespecified observation period. No interim analysis of the primary outcome will be performed, and the observation period will not be modified on the basis of accumulating results. The numbers of analyzable administrations, contributing professionals, and observation sessions will be reported by center. If the planned number is not reached in either group, the power loss will be reported and comparative analyses interpreted as exploratory.

The professional sample of approximately 40 participants is a feasibility estimate, not a UEQ sample-size calculation; UEQ analyses will therefore emphasize estimation; scale-reliability estimates, particularly in small technology groups, will be treated as exploratory and reported only when estimable.

#### Eligibility of administrations and data sources

An eligible administration will be an intravenous antineoplastic infusion performed in routine practice by a consenting nursing professional during a prespecified data-collection period, with sufficient information to determine the technology used and the primary outcome. Actual follow-up duration and completion status will be recorded. Administrations with incomplete follow-up and no recorded air-in-line alarm will not be classified as event-free; such outcomes will be unascertained, time-to-first-alarm follow-up will be censored, and a sensitivity analysis will be restricted to complete follow-up.

#### Administration-level variables and outcomes

The primary outcome will be the occurrence of at least one air-in-line alarm during an eligible administration.

Secondary outcomes will include the total number of air-in-line alarms; occurrence and type of other alarms; time to first alarm; alarm-management time; infusion interruption; alarm recurrence; maintenance or loss of a closed circuit; management strategies; manipulation or replacement of the administration set; and restoration of infusion continuity.

Alarm-management time will be defined as the interval from alarm activation or documented recognition to safe resumption of the prescribed infusion; scheduled pauses, end-of-infusion signals, planned bag changes, and interruptions unrelated to the infusion system will be excluded.

Prespecified administration characteristics will include center, infusion technology, drug or regimen category, foaming or outgassing classification (finalized before outcome analysis using pharmaceutical documentation and expert review), programmed flow rate, planned infusion duration, administration-set characteristics, and available device settings.

#### User experience questionnaire

Healthcare professionals’ user experience with each eligible infusion technology will be assessed using the official Italian-language version of the 26-item User Experience Questionnaire (UEQ) [25].

The UEQ comprises six scales (Attractiveness, Perspicuity, Efficiency, Dependability, Stimulation, Novelty); its 26 bipolar seven-point items are scored from −3 to +3 according to the official handbook, and no overall total score will be created.

Each professional will complete a separate UEQ for each technology with which they have sufficient exposure, defined as routine use and involvement in at least three eligible administrations with that technology during the study period.

The UEQ does not directly measure alarm clarity, ease of alarm resolution, perceived clinical safety, or operational workload. If these aspects are investigated, study-specific supplementary items will be placed in a separate section after the complete, unmodified UEQ and reported separately from UEQ scale scores.

#### Data quality

Automated and manual checks will assess the consistency of alarm occurrence, count, type, management time, interruption, recurrence, and completion status. Queries will be resolved against source information before database lock, and corrections will preserve an auditable record of the original value, reason, date, and responsible person.

#### Statistical analysis

Administration-level and professional-level data will initially be analyzed separately.

Alarm frequencies will be reported as counts, proportions, and 95% confidence intervals; counts and management times as means and standard deviations or medians and interquartile ranges; and time to first alarm will treat administrations without an alarm as right-censored at infusion completion or the end of available follow-up. The primary unadjusted comparison will estimate the risk difference, risk ratio, and odds ratio for at least one air-in-line alarm, with 95% confidence intervals. Between-technology comparisons will describe differences between the observed technology–center configurations and will not isolate technology from center effects. Interval estimation will account for repeated administrations within professionals where estimable; uncertainty will be conditional on the participating centers and will not quantify between-center variation for Device A. Any independence-based intervals used because clustered estimation is unstable will be explicitly identified as nominal.

Adjusted associations with binary alarm outcomes will be examined using mixed-effects logistic regression, with center as a fixed effect, a professional-level random intercept, and, where identifiable, pump-unit clustering; model complexity will be limited by the number of events. Alarm counts may be analyzed using Poisson or negative-binomial regression, and management time modeled, only if events are sufficient. Candidate covariates will be selected before analysis (drug or regimen category, foaming classification, planned duration, flow rate, professional experience, device or set characteristics). Models including center as a fixed effect will examine covariates with sufficient within-center variation; a separate technology coefficient will not be estimated in these models.

Adjusted analyses will estimate associations rather than causal effects. Given the alignment of technology with center in Phase 3, propensity-score adjustment will not be performed for the comparison of Device A with Devices B and C.

UEQ scores will be calculated separately for Attractiveness, Perspicuity, Efficiency, Dependability, Stimulation, and Novelty. Each scale will be summarized using its mean, standard deviation, 95% confidence interval, and, where appropriate, comparison with the official UEQ benchmark [26]. Internal consistency will be evaluated separately for each scale using Cronbach’s alpha and, where estimable, McDonald’s omega.

If the same professionals evaluate more than one technology, comparisons will use paired or mixed-effects methods; otherwise, between-group UEQ comparisons will describe the observed technology–center configurations, with adjustment for professional experience and exposure where feasible; they will not be interpreted as technology effects independent of center. Comparisons across the six UEQ scales will be secondary, with multiplicity controlled by the Holm procedure or explicitly labeled exploratory.

Missingness will be reported for every outcome, covariate, and UEQ scale. Missing primary outcomes will not be imputed; UEQ responses will follow official scoring guidance; multiple imputation may be used for covariates only when its assumptions are supported, with comparison against complete-case analyses.

All tests will be two-sided, with p < 0.05 used as the nominal threshold for the prespecified primary comparison within the observed configurations; secondary and exploratory analyses will emphasize effect estimates, confidence intervals, multiplicity, model stability, and clinical relevance.

## Study timeline

The overall estimated duration of the study is approximately 24 months from CEN approval (July 20, 2026) and completion of local authorization procedures, organized into three sequential, partially overlapping phases:

**Table:** 

Activity	M1–3	M4–6	M7–9	M10–12	M13–15	M16–18	M19–21	M22–24
Study setup and ethics approval	*Done*							
Phase 1 - Simulation study	■	■						
Phase 1 data analysis			■					
Phase 2 - Field observational study			■	■				
Phase 2 data analysis					■			
Phase 3 - Clinical observational study					■	■	■	
Final data analysis							■	■
Publication and dissemination						■	■	■

## Data management

Data will be collected with phase-specific standardized forms in REDCap (Research Electronic Data Capture) [27], which ensures secure storage, traceability of changes, and data integrity. Data will be recorded in pseudonymized form, analyzed only in aggregate, and accessible only to authorized personnel through individual credentials and role-based permissions. Periodic data-quality and consistency checks will be conducted.

## Infusion devices

All infusion devices included in the study are commercially available, CE-marked medical devices, used exclusively within their intended purpose and in accordance with the manufacturer’s instructions for use and routine local practice. The study will not require any modification of devices, infusion sets, software, alarm systems, or operating configurations.

The study will compare one cassette-based, piston-actuated positive-displacement volumetric infusion system with two tubing-based linear-peristaltic volumetric infusion systems used as comparators (Table 1), representing the technologies routinely available at the participating centers. Device allocation will not be determined by the investigators.

Manufacturer, exact model, software version, physical pump-unit identifier, compatible administration set, air-in-line and alarm settings, and maintenance or calibration status will be documented before data collection and reported in a supplementary device-identification table; generic technological descriptors will be used in comparative analyses.

Devices B and C are different commercial models sharing the same core operating principle and will be pooled a priori in the primary analyses. Pooling does not imply technical or clinical equivalence: device-specific characteristics and descriptive results will be reported, and sensitivity analyses will describe differences between Devices B and C if observations and events are sufficient, without separating device effects from center effects when these are aligned.

## Ethics, consent, and data protection

Phase 1 will generate exclusively technical data. Phases 2 and 3 will involve the processing of common personal data of participating healthcare professionals, limited to sex, age group, years of professional experience in prespecified ranges, UEQ responses, and structured observations of professional behaviors and strategies. No special categories of personal data will be collected.

Before participation, healthcare professionals will receive a study information document and the information required under Article 13 of Regulation (EU) 2016/679, and will provide written informed consent to study participation and separate consent to the processing of personal data. Refusal or withdrawal will have no consequences for employment, professional evaluation, training opportunities, or the relationship with the institution.

Data minimization and coding will be applied at source: direct identifiers will not be entered into REDCap, consent forms will be stored separately without any linkage code, and no identification key will be created or transferred to the Promoter. A documented phase-specific coding procedure will enable repeated observations and withdrawal requests to be linked to the relevant records before irreversible anonymization or aggregation. The procedure for issuing and retaining codes will be specified in the participant information and observer manual. Individual-level results will not be communicated to supervisors or used for any employment-related decision, and reports will be produced at group level with small cells suppressed or aggregated.

Patients receiving the observed administrations will not be enrolled as study participants, and no patient assessment will be performed; observers will not interact with patients, influence clinical decisions, delay therapy, or modify the prescribed infusion. Authorized clinical personnel will consult the therapy chart only to extract the minimum non-identifiable information needed to characterize the infusion (e.g., categorized drug or preparation type, infusion configuration, flow parameters, alarm characteristics). No patient names, identification codes, medical-record numbers, dates of birth, diagnoses, clinical outcomes, or exact dates or clock times permitting identification will be entered into the database.

Each institution will process data under the GDPR and Italian legislation, with documented roles for centers, Promoter, and data processors. The dataset will be retained for 10 years after study completion. Participants may withdraw consent at any time without affecting the lawfulness of prior processing; data already anonymized or aggregated may no longer be removable.

## Benefits and risks for participants

No direct benefit is expected for participants. Foreseeable risks are minimal (time burden, discomfort from being observed, limited confidentiality or reputational risk) and will be mitigated through data minimization, source coding, restricted access, aggregate reporting, and the prohibition of individual performance evaluation; insurance and liability requirements will follow national legislation and Ethics Committee conditions.

## Dissemination of research results

Findings will be disseminated through peer-reviewed publications and conference presentations irrespective of their direction or statistical significance, exclusively in aggregate form; no professional, patient, or center will be identifiable in comparative rankings unless expressly authorized. Authorship eligibility will follow ICMJE criteria, and author contributions will be described using the CRediT taxonomy. The funder’s role and conflicts of interest will be fully disclosed.

Where compatible with consent, ethics approval, and data protection, the protocol, data dictionaries, instruments, statistical code, and sufficiently anonymized data may be shared through a repository once the risk of indirect identification has been excluded.

## Protocol validation

The protocol underwent methodological, ethical, and operational review by investigators with expertise in oncology nursing, infusion therapy, simulation, medical devices, human factors, research methodology, biostatistics, and data protection. Preliminary calibration and pilot runs conducted by AIIAO supported the technical feasibility of the simulation procedures, the practicability of data collection, and the plausibility of the planning proportions, which will not be treated as confirmatory estimates.

Before full implementation, all study-specific instruments will undergo content validation by a multidisciplinary panel of at least six experts in oncology nursing, infusion therapy, clinical research methodology, medical-device technology, simulation, and human factors, who will independently rate the relevance, clarity, completeness, feasibility, and neutrality of each item and operational definition. Relevance will be rated on a four-point scale; ratings of 3 or 4 will count as agreement on relevance, while the other domains will inform item revision. Content validity will be quantified using the item-level Content Validity Index (I-CVI) and the average scale-level index (S-CVI/Ave), with prespecified acceptability criteria of I-CVI ≥ 0.78 and S-CVI/Ave ≥ 0.90 [28,29]. Items not meeting these criteria or found ambiguous will be revised and reassessed, and the instrument version history will be documented.

For Phase 1, calibration runs will verify the reproducibility of preparation handling, priming, set configuration, flow rate, environmental conditions, device programming, alarm settings, and outcome timing; formal simulations will begin only when the operating procedure can be implemented consistently and all variables captured unambiguously.

For Phase 2, the EPA-informed observation grid will be pilot-tested in scenarios excluded from the main analysis, supported by an observer manual defining each behavior, event, timing start and end point, and adherence criterion. Trained observers will independently score the same pilot scenarios. A Cohen’s kappa (or an appropriate extension) ≥ 0.70 is the protocol-defined criterion for categorical variables. For continuous measures, including alarm-management time, a two-way random-effects, absolute-agreement, single-measure intraclass correlation coefficient (ICC[2,1]) will be used, with a protocol-defined threshold of ≥ 0.75 [30]. Estimates will be reported with 95% confidence intervals and interpreted in relation to the number of scenarios and raters. If the point-estimate criteria are not met, definitions will be clarified, observers retrained, and agreement reassessed; periodic duplicate observations will monitor observer drift.

For Phase 3, the data-collection form will undergo cognitive and operational pretesting with representative professionals (comprehension, burden, completion time, workflow fit); pilot records will be excluded from analysis.

The REDCap database will undergo user-acceptance testing with predefined valid, boundary, missing, and inconsistent test cases before activation, and data collection will start only after all critical discrepancies have been resolved. The data dictionary, observer manual, simulation standard operating procedure, and device-characterization form will be finalized before the corresponding main data collection begins. The statistical analysis plan will be finalized before examination of main-study outcome data for analysis, and analysis code will be tested on simulated or pilot datasets.

Protocol adherence, recruitment, missingness, interobserver agreement, and completeness of device documentation will be monitored throughout; deviations will be documented and considered in sensitivity analyses, and substantive modifications will be version-controlled and submitted for approval when required.

## Limitations

Phase 1 is conducted under controlled simulation conditions that may not reproduce the complexity of real oncology practice; its results should not be interpreted as estimates of clinical effectiveness or patient outcomes.

In Phases 2 and 3, the observational design precludes causal inference, residual confounding may persist despite adjustment, and consecutive inclusion during predefined periods may introduce selection or temporal bias if those periods are not representative.

Infusion technology is fully aligned with center: the cassette-based system is in routine use at a single center and the tubing-based systems at the remaining four. The Device A versus pooled Devices B/C contrast is therefore completely confounded with center, and no analytical strategy can identify a technology effect separately from center. Comparisons will describe the observed configurations. The a priori pooling of Devices B and C, based on their shared operating principle, does not imply technical or performance equivalence.

Clustering of administrations within professionals and centers may reduce effective sample size and precision. Center fixed effects can support analyses of covariates that vary within centers, but cannot identify a separate technology effect under complete alignment. With one center using Device A, between-center variability for that technology cannot be estimated. Dependence among repeated administrations received by the same patient cannot be identified or directly modeled because patient identifiers will not be collected; this may further affect precision.

Structured observation may be affected by the Hawthorne effect, observer subjectivity, and interobserver variability, mitigated through standardized definitions, observer training, pilot testing, and agreement assessment. Because the grid is EPA-informed but study-specific, its measurement properties will require cautious interpretation.

The UEQ is a generic self-reported measure and does not provide objective measures of device safety, alarm-management performance, or workload; its results may be influenced by prior experience, exposure, expectations, and social desirability. Because Phases 2 and 3 will not be linked at the professional level, associations between UEQ scores and observed adherence across those phases will not be estimated. Finally, differences in device model, software version, infusion set, air-detection configuration, maintenance status, preparation characteristics, and local procedures may influence alarm occurrence and management; these will be documented and considered in analyses where feasible. Because no patient-level outcomes will be collected, the study cannot determine whether observed differences affect morbidity or treatment effectiveness.

## Related research article

None.

## Protocol overview

• Conduct a controlled simulation study to compare infusion technologies under standardized conditions.
• Observe routine oncology infusion practices to describe professional practices and strategies used to manage air-in-line alarms.
• Collect prospective multicenter real-world data on alarm frequency, management, and healthcare professionals’ user experience to inform evidence-based recommendations.

## CRediT author statement

Rosario Caruso: Conceptualization, Methodology, Project administration, Supervision, Writing – original draft, Writing – review & editing. Greta Ghizzardi: Conceptualization, Methodology, Project administration, Investigation, Writing – original draft, Writing – review & editing. Tatiana Bolgeo: Methodology, Validation, Writing – review & editing. Monica Liberatori: Investigation, Resources, Project administration, Writing – review & editing. Daniele Napolitano: Investigation, Resources, Project administration, Writing – review & editing. Anna Carmagnola: Investigation, Resources, Project administration, Writing – review & editing. Andrea Moro: Investigation, Resources, Project administration, Writing – review & editing. Ilaria Milani: Conceptualization, Methodology, Validation, Writing – review & editing. Stefano Maiandi: Investigation, Resources, Project administration, Writing – review & editing. Gianluca Conte: Methodology, Validation, Writing – review & editing. Marco Alfredo Arcidiacono: Investigation, Resources, Project administration, Writing – review & editing. Serena Caponetti: Methodology, Validation, Writing – review & editing. Gabriele Cremona: Methodology, Validation, Writing – review & editing. Marcella Dabbene: Methodology, Validation, Writing – review & editing. Viviana Riccobene: Investigation, Resources, Writing – review & editing. Monica Guberti: Methodology, Validation, Writing – review & editing. Alessio Piredda: Investigation, Resources, Project administration, Writing – review & editing. Arianna Magon: Conceptualization, Methodology, Validation, Writing – review & editing.

## Declaration of competing interest

The authors declare the following financial interests/personal relationships which may be considered as potential competing interests: This study received unrestricted support from ICU Medical, Inc., which manufactures one of the infusion systems evaluated in this protocol. The support was provided to the Italian Association of Oncology Nurses (Associazione Italiana Infermieri di Area Oncologica, AIIAO), which is responsible for the scientific coordination of the study. No author received any payment, honorarium, consultancy fee, or other personal financial benefit from ICU Medical, Inc., either directly or through their employing institutions, in connection with this work or outside it. The funder had no role in the study design or protocol development and will have no role in data collection, data management, statistical analysis, interpretation of the results, manuscript preparation, or the decision to submit the findings for publication. The protocol prespecifies that findings will be reported irrespective of their direction or statistical significance.

## Data Availability

No data was used for the research described in the article.
